# Supplementary material for: Hell’s Gate Globin-I from Methylacidiphilum infernorum Displays a Unique Temperature-Independent pH Sensing Mechanism Utililized a Lipid-Induced Conformational Change
Source: Int J Mol Sci. 2024 Jun 20;25(12):6794. doi: 10.3390/ijms25126794 (PMC11203436; doi:10.3390/ijms25126794)
Supplement: Supplementary file 1 [file ijms-25-06794-s001.zip › ijms-3008050-supplementary.pdf]

Supporting Information

Hell's Gate Globin-I from *Methylacidiphilum infernorum* Displays a Unique Temperature-Independent pH Sensing Mechanism Utililized a Lipid-Induced Conformational Change

Brandon J. Reeder * , Dimitri A. Svistunenko and Michael T. Wilson

School of Biological Sciences, University of Essex, Wivenhoe Park Colchester, Essex CO4 3SQ, UK;

svist@essex.ac.uk (D.A.S.); wilsmt@essex.ac.uk (M.T.W.)

* Correspondence: reedb@essex.ac.uk; Tel.: +44-1206-872119

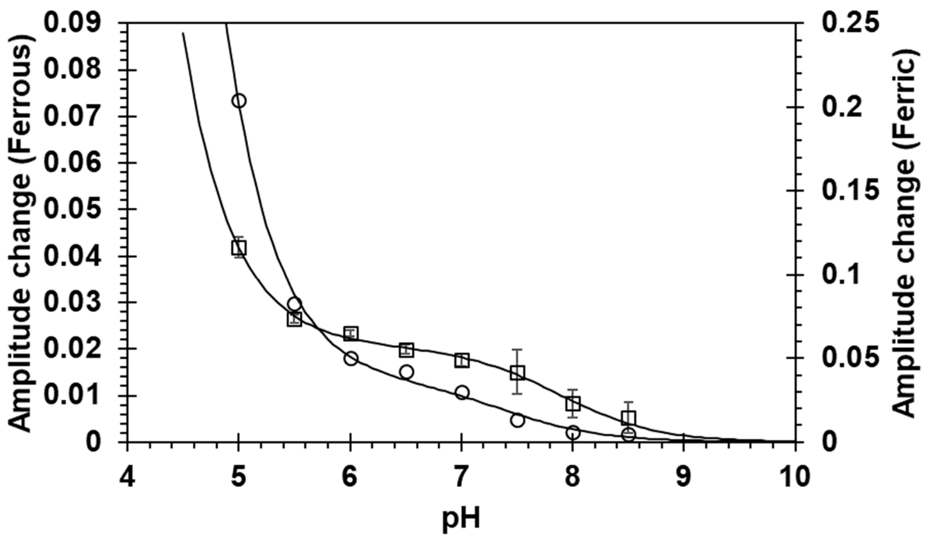

Figure S1. Amplitude changes observed for the pH jump from pH 10 at 416 nm (Ferrous, ☐) and 400 nm (Ferric, ○).

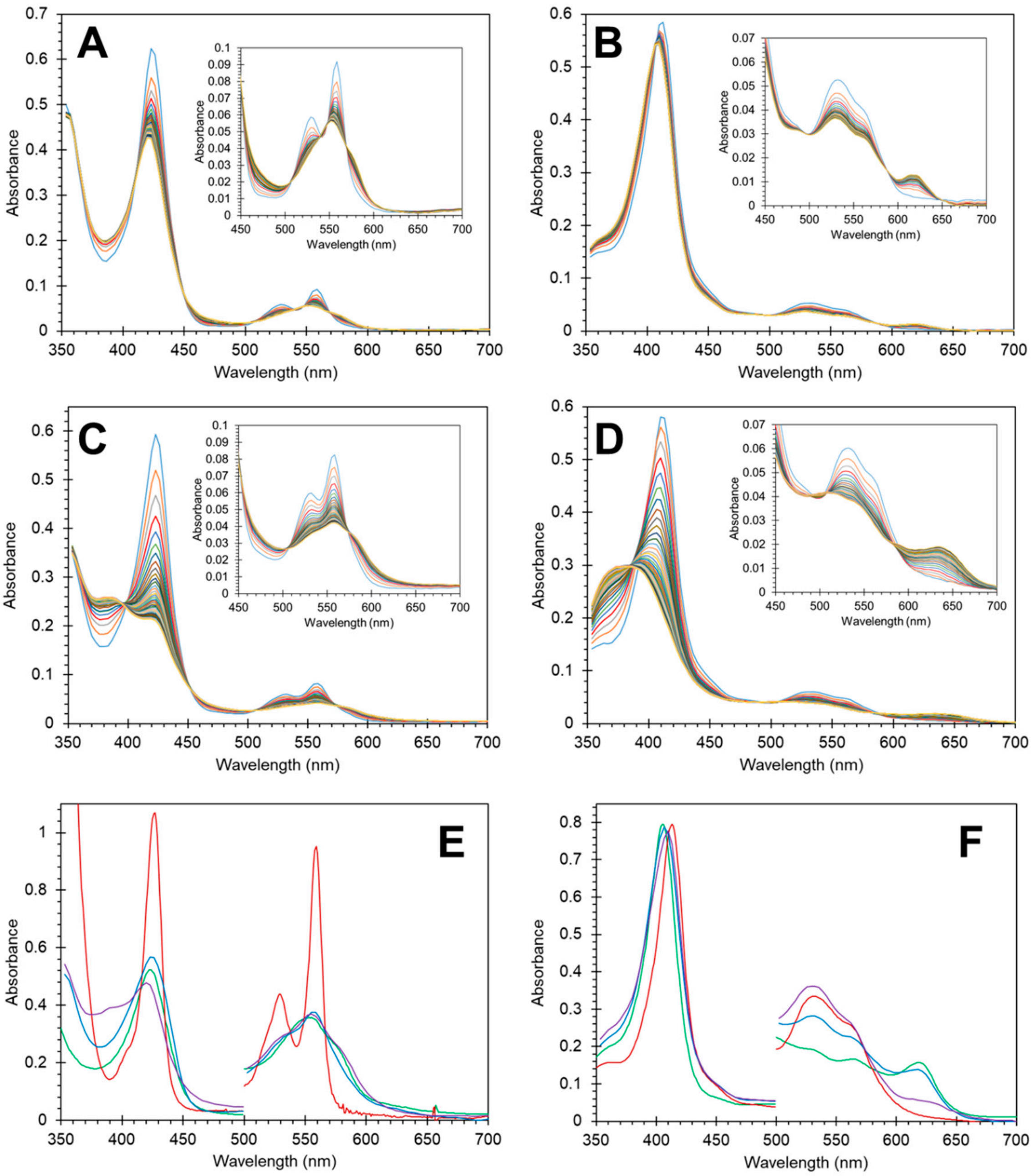

Figure S2. Effects of pH on optical spectra of ferrous (A) and ferric (B) HGb-I. Optical changes following pH jump from 10 to 3.5 for ferrous HGb-I (A) and for ferric HGb-I (B). Optical changes following pH jump from 10 to 2.5 for ferrous HGb-I (C) and for ferric HGb-I (D). (E) Ferrous spectra at pH 10 (red) and pH 5 (green) taken on a scanning spectrophotometer. Stopped-flow pH jump spectra at optimal pentacoordinate form to pH 5 (blue) and pH 2.5 (purple). (F) As for spectra in (E) except with ferric protein.

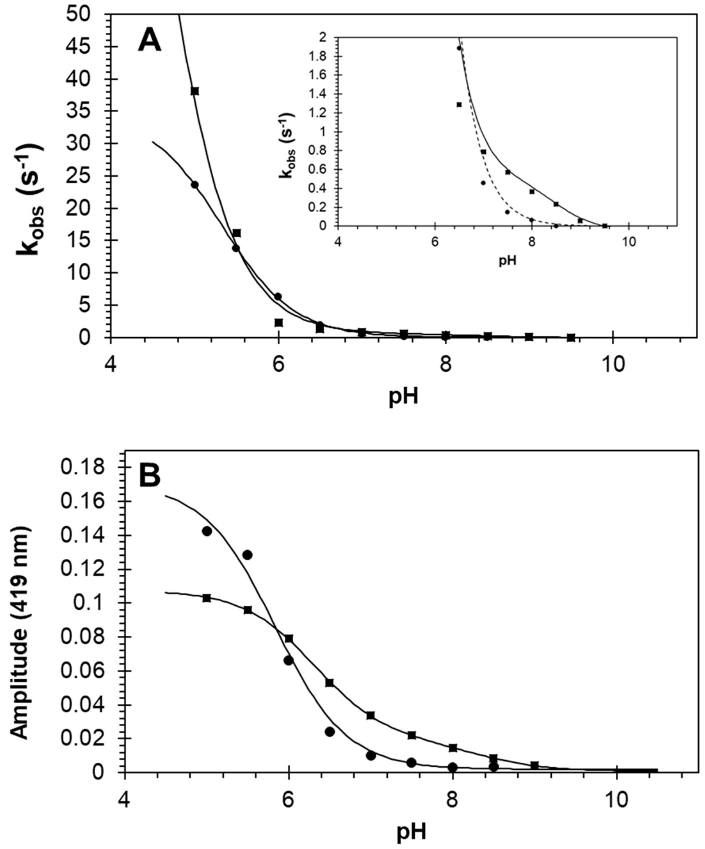

Figure S3. Effect of pH and oleate on the binding of azide to ferric HGb-I. (A) Rate constant for azide binding to HGb-I in the absence (●) and presence (■) of five-fold excess sodium oleate. The observed rate in the absence of oleate exhibited a pK of 5.45±0.05 and a k_{max} 32.5s^{-1}±1.43. In the presence of oleate this changed to a projected pK of 4.5 and k_{max} ~267, although the error margins with these values are considerable. Inset: Expanded view. (B) Amplitude change for azide binding to HGb-I in the absence (●) and presence (■) of five-fold excess sodium oleate. The amplitude change in the absence of oleate exhibited a pK of 5.83±0.10 and an amplitude change (419 nm) of 0.171±0.011. In the presence of oleate this changed to a pK of 6.31±0.07 and an amplitude change (419 nm) of 0.089±0.003, there is a small secondary pK at 8.30 with an amplitude of 0.0184.

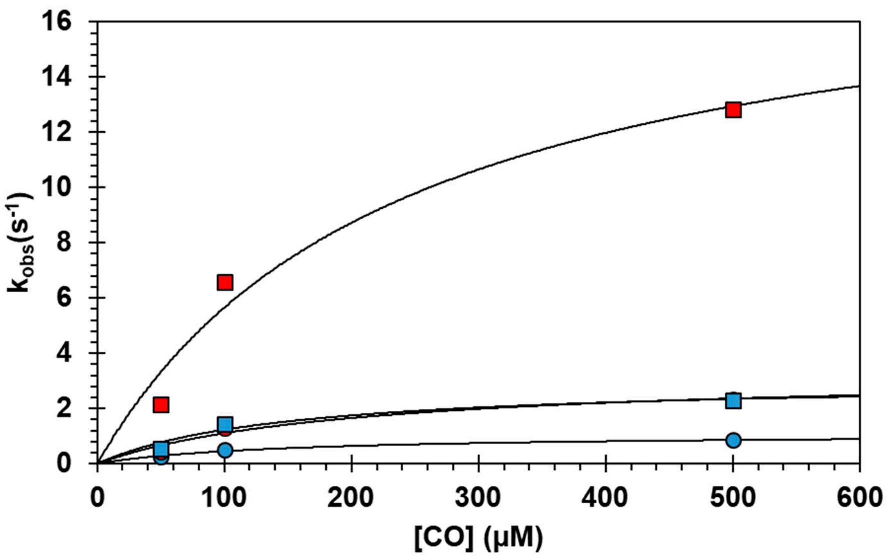

Figure S4. Effect of pH and oleate on the binding of carbon monoxide to ferrous HGb-I. Rate constant for CO binding to HGb-I in the absence (●) and presence (■) of five-fold excess sodium oleate. Red is pH 8.5 and blue pH 10. Experiments at pH 5 did not show CO binding to protein. Maximum rate constants were 1.1 and 3.3 s^{-1} for pH 10 and 8.5 respectively (without oleate) and 3.0 and 19.1 s^{-1} for pH 10 and 8.5 respectively (with oleate). K_D was 143 μM (without oleate) and 145 μM (with oleate) CO for pH 10. K_D was 199 μM (without oleate) and 237 μM (with oleate) CO for pH 8.5.

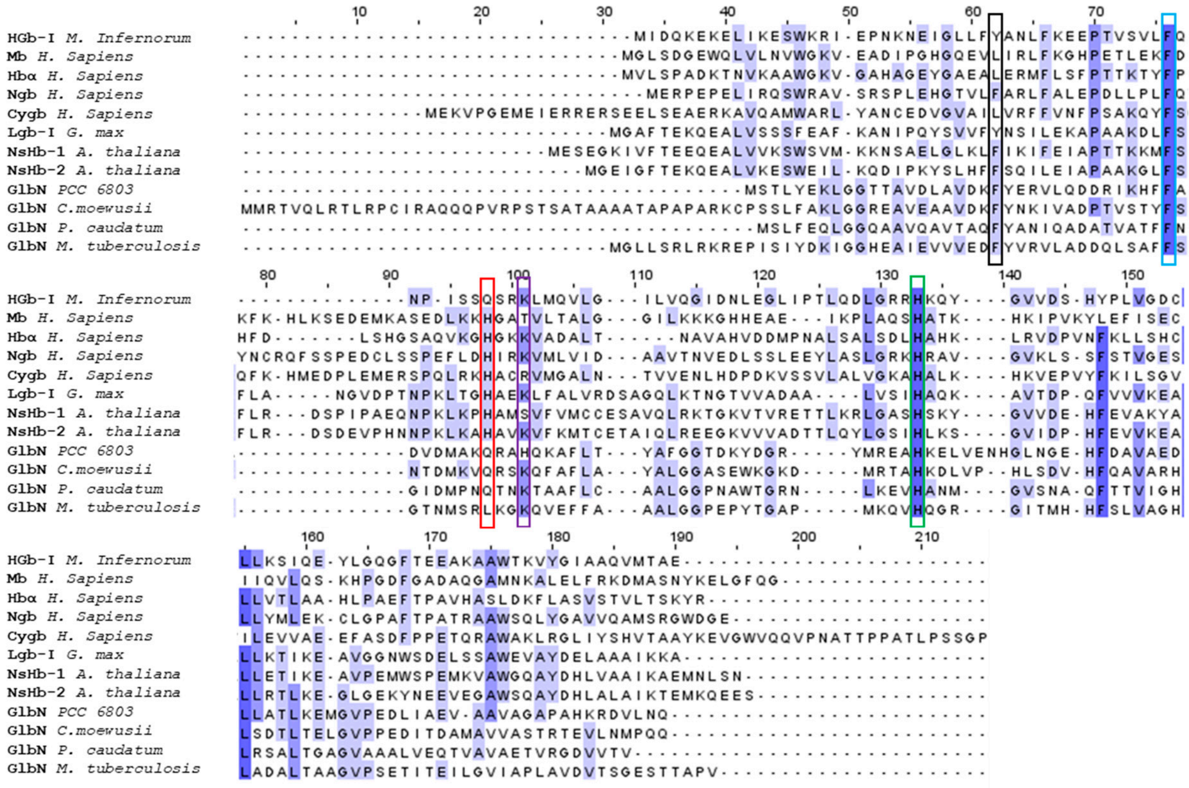

Figure S5. Sequence alignment of HGb-I with selected human, plant and bacterial globins. HGb-I was sequence aligned using Muscle on Jalview v1.8.3 and all sequences were obtained through Uniprot. Sequences are human myoglobin (Mb), human hemoglobin alpha chain (Hbα), human neuroglobin (Ngb), human Cytoglobin (Cygb), soybean leghemoglobin (Lgb-I), Arabidopsis non-symbiotic hemoglobin (phytoglobin) class 1 (NsHb-1) and class 2 (NsHb-2), and truncated globins (GlbN) from *Synechocystis sp. PCC 6803, C.moewusii, P. caudatum* and *M. tuberculosis*. Blue gradient denotes amino acids with high sequence percentage identity. Boxes highlight specific amino acids B10 (black box) CD1 (cyan box), E7 (red box, distal heme ligand for most), E10 (purple box) and proximal heme ligand F8 (green box).

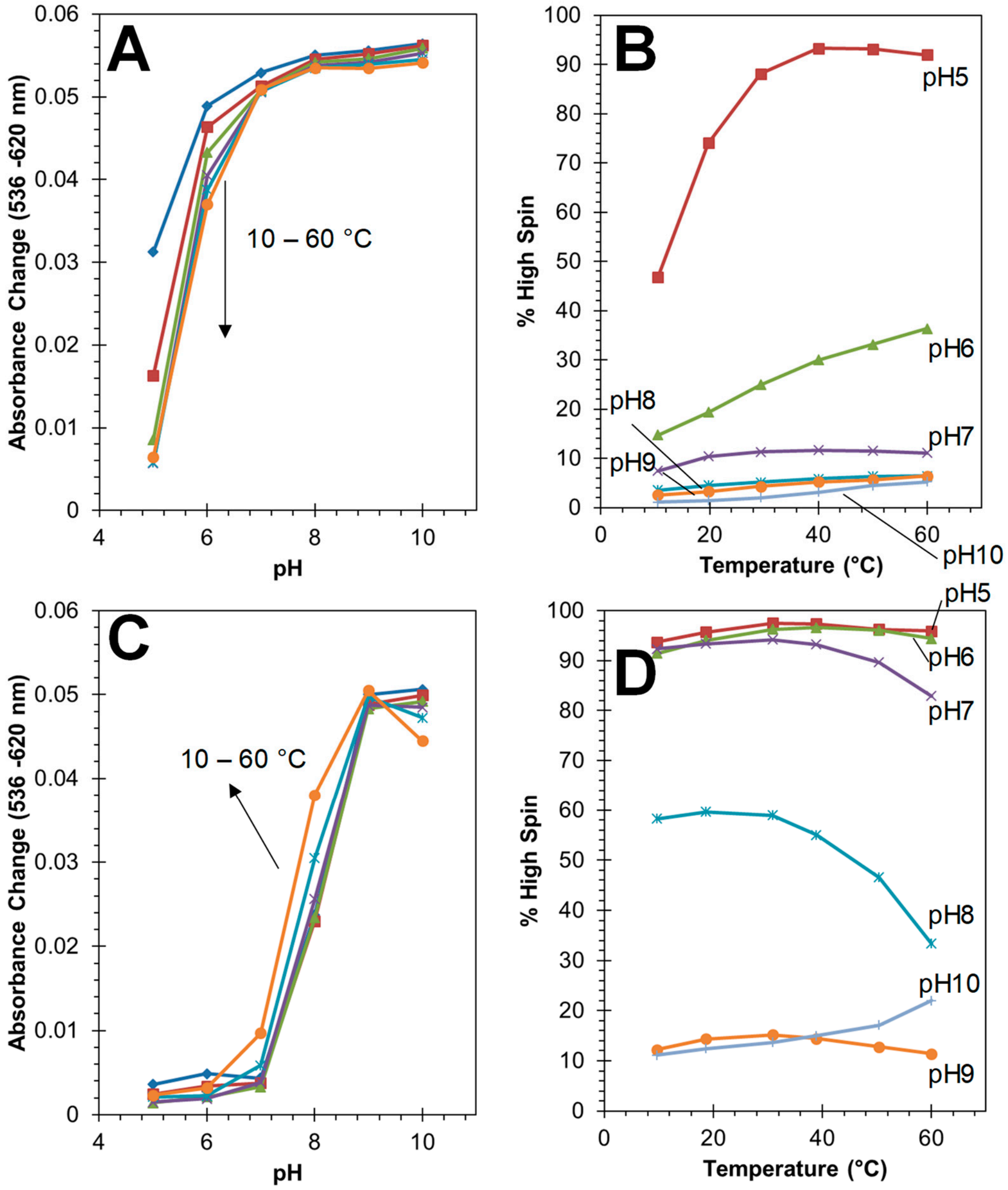

Figure S6. Effect of temperature and pH on the coordination state of ferric HGb-I. (A) Optical changes resulting in heme iron co-organization state changes for ferric HGb-I (B) Data from (A) presented as a function of temperature. (C) (A) Optical changes resulting in heme iron co-organization state changes for ferric HGb-I in the presence of 5 molar excess oleate (D) Data from (C) presented as a function of temperature. % High Spin was calculated based on the absorbance changes of the protein at pH 10 (in absence of oleate) for a fully hexacoordinated, low-spin protein) and pH 5 (in presence of oleate) for a fully pentacoordinate, high-spin protein). Buffers were as those described in Figure 2.

Table S1. Ligand and lipid binding affinities, lipid oxidation, heme iron coordination and redox potential comparison of selected globins.

	**HGb-I**	**Mb**	**Cygb**	**Ngb**	**trHbN**
Oxygen affinity (μM)	0.53 (25 °C) [5]<br>6.8 (50 °C) [5]	1.2 [55]	0.2 [56]	0.032 [53]	0.008 [51]
Lipid affinity (μM)	0.73 ± 0.03<br>(oleate, 20 °C) This work	43 (parmitate, 35 °C) [58]<br>45 (oleate, 35 °C) [57]	0.70 (oleate, 25 °C) [18]	Negligible [18]	ND
Maximum Liposome Oxidation Rate (nM s^{-1})	Negligible	8.36 ± 2.26 [18]	44.1 ± 6.25 [18]	Negligible [28]	ND
*Ferrous heme geometry<br>(Spin state)	Hexacoordinate<br>(LS)	Pentacoordinate<br>(HS)	Hexacoordinate<br>(LS)	Hexacoordinate<br>(LS)	Pentacoordinate<br>(HS)
Ferrous heme geometry*<br>+ Lipid<br>(Spin state)	Pentacoordinate<br>(HS)	Pentacoordinate<br>(HS)	Hexacoordinate<br>(LS)	Hexacoordinate<br>(LS)	ND
*Ferric heme geometry<br>(Spin state)	Hexacoordinate<br>(LS)	Pentacoordinate<br>(HS)	Hexacoordinate<br>(LS)	Hexacoordinate<br>(LS)	Pentacoordinate<br>(HS)
*Ferric heme geometry+ Lipid<br>(Spin state)	Pentacoordinate<br>(HS)	Pentacoordinate<br>(HS)	Pentacoordinate<br>(HS)	Hexacoordinate<br>(LS)	ND
*Redox Potential (mV)	−305 [59]	−160 [54]	−189 [52]	-118 [60]	ND

*Data obtained in absence of ligand, neutral pH.

Further exploration of the protonation-coordination change models.

Model 1:

A site on the hexacoordinate heme protein P_h is getting protonated and the protonated form finds itself in a reversible equilibrium with a penta-coordinate heme state P_pH:

$$P_h \underset{-H^+}{\overset{+H^+}{\rightleftarrows}} P_hH \underset{k_b}{\overset{k_f}{\rightleftarrows}} P_pH \qquad \text{(S1)}$$

We can write down a system of three equations:

$$\left.\begin{aligned} &[P_h] + [P_hH] + [P_pH] = P_h^0 && \text{(S2)} \\ &\frac{d[P_pH]}{dt} = k_f[P_hH] - k_b[P_pH] && \text{(S3)} \\ &K_A = \frac{[P_h][H^+]}{[P_hH]} && \text{(S4)} \end{aligned}\right\} \text{System 1}$$

From equation (S4) of System 1,

$$[P_h] = \frac{K_A[P_hH]}{[H^+]}$$

We substitute this to equation (S2) of the system:

$$\frac{K_A[P_hH]}{[H^+]} = P_h^0 - [P_hH] - [P_pH]$$

or

$$[P_hH]\left(\frac{K_A}{[H^+]} + 1\right) = P_h^0 - [P_pH]$$

which gives

$$[P_hH] = \frac{P_h^0 - [P_pH]}{\frac{K_A}{[H^+]} + 1}$$

Thus, we can re-write equation (S3) in System 1 that gives the rate of the pentacoordinate (protonated within our model) state formation, which can be followed spectroscopically:

$$\frac{d[P_pH]}{dt} = k_f \frac{P_h^0 - [P_pH]}{\frac{K_A}{[H^+]} + 1} - k_b[P_pH]$$

or

$$\frac{d[P_pH]}{dt} = k_f \frac{P_h^0}{\frac{K_A}{[H^+]}+1} - k_f \frac{[P_pH]}{\frac{K_A}{[H^+]}+1} - k_b[P_pH]$$

Or

$$\frac{d[P_pH]}{dt} = \underbrace{k_f \frac{P_h^0}{\frac{K_A}{[H^+]}+1}}_{A} - \underbrace{(k_f \frac{1}{\frac{K_A}{[H^+]}+1} + k_b)}_{k}[P_pH] \quad \text{(S5)}$$

Equation (S5) is a type of a

$$\frac{dy}{dt} = A - ky \quad \text{(S6)}$$

equation (where $y = [P_pH]$). We can transform it as follows:

$$\frac{1}{k}\frac{dy}{dt} = \frac{A}{k} - y$$

If we further define a new variable z as

$$z = y - \frac{A}{k} \quad \text{(S7)}$$

Equation (S6) is turning into

$$\frac{1}{k}\frac{d(z+\frac{A}{k})}{dt} = -z$$

or, finally,

$$\frac{dz}{dt} = -kz \quad \text{(S8)}$$

…which is an exponential decay with a rate constant *k*.

Since from (S7) it follows that

$$\frac{dz}{dt} = \frac{dy}{dt}$$

and, as was defined in (S6), $y = P_pH$,

constant *k* describes apparent decay of penta-coordinate form (protonated) P_pH.

Thus, apparent rate constant of formation of P_pH, as a function of proton concentration, should be described by k, defined in (S5) as a function of [H$^+$], i.e.

$$k_{apparent} = k_f \frac{1}{\frac{K_A}{[H^+]} + 1} + k_b \quad \text{(S9)}$$

Thus this rate constant has two limit values:

$$\lim_{[H^+]\to 0} \left(k_f \frac{1}{\frac{K_A}{[H^+]} + 1} + k_b \right) = k_b$$

and

$$\lim_{[H^+]\to \infty} \left(k_f \frac{1}{\frac{K_A}{[H^+]} + 1} + k_b \right) = k_f + k_b$$

$k_f + k_b$ is greater than k_b

So, as $[H^+]$ increases from 0 to infinity, we should have

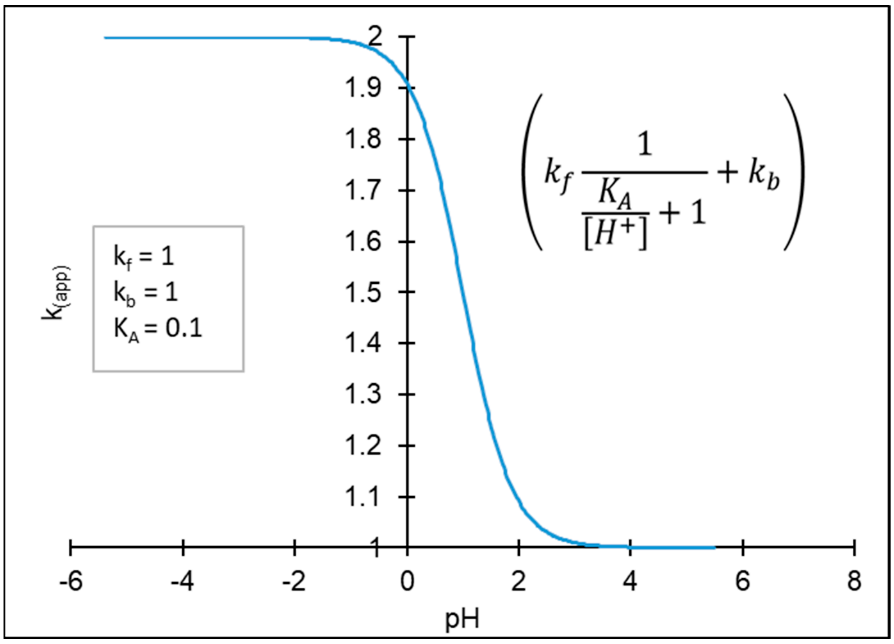

Model 2:
Hexa- and penta-coordinate heme forms of the protein are in equilibrium, but it is the penta-coordinate heme form that can be protonated to give the P_pH form that we detect optically:

$$P_h \underset{k_b}{\overset{k_f}{\rightleftarrows}} P_p \underset{-H^+}{\overset{+H^+}{\rightleftarrows}} P_pH \quad \text{(S10)}$$

In a way similar to our description of Model 1, we would like to define the rate of the experimentally detected P_pH formation, $d[P_pH]/dt$.

First, the sum of the three forms of the proteins should stay constant:

$$[P_h] + [P_p] + [P_pH] = P_h^0 \tag{S11}$$

Further, the equilibrium constant K_A of the protonation/deprotonation of the penta-coordinate form

$$K_A = \frac{[P_p][H^+]}{[P_pH]}$$

allows expressing of P_p as function of P_pH and H^+:

$$[P_p] = \frac{K_A[P_pH]}{[H^+]} \tag{S12}$$

Expression (S12) can be substituted to (11):

$$[P_h] + \frac{K_A[P_pH]}{[H^+]} + [P_pH] = P_h^0$$

or

$$[P_h] = P_h^0 - (\frac{K_A}{[H^+]} + 1)\ [P_pH] \tag{S13}$$

or

$$[P_pH] = \frac{P_h^0 - [P_h]}{\frac{K_A}{[H^+]} + 1}$$

or

$$[P_pH] = \frac{P_h^0}{\frac{K_A}{[H^+]} + 1} - \frac{[P_h]}{\frac{K_A}{[H^+]} + 1} \tag{S14}$$

Now, the derivative

$$\frac{d[P_pH]}{dt}$$

can be expressed as

$$\frac{d[P_pH]}{dt} = -\frac{1}{\frac{K_A}{[H^+]}+1}\frac{d[P_h]}{dt} \tag{S15}$$

We have to express $d[P_h]/dt$ and substitute it to (S15) to have $d[P_pH]/dt$ as a function of [H^+] only. From equations (S10) and (S12), it follows that

$$\frac{d[P_h]}{dt} = -k_f[P_h] + k_b[P_b] = -k_f[P_h] + k_b\frac{K_A[P_pH]}{[H^+]}$$

We substitute this to (15) :

$$\frac{d[P_pH]}{dt} = -\frac{1}{\frac{K_A}{[H^+]}+1}\frac{d[P_h]}{dt} = -\frac{1}{\frac{K_A}{[H^+]}+1}(-k_f[P_h] + k_b\frac{K_A[P_pH]}{[H^+]})$$

And substituting [P_h] from (S13)

$$\frac{d[P_pH]}{dt} = -\frac{1}{\frac{K_A}{[H^+]}+1}(-k_fP_h^0 + k_f(\frac{K_A}{[H^+]}+1)\,[P_pH]) + k_b\frac{K_A[P_pH]}{[H^+]}) =$$

$$= \frac{k_fP_h^0}{\frac{K_A}{[H^+]}+1} - \frac{k_f(\frac{K_A}{[H^+]}+1)\,[P_pH]}{\frac{K_A}{[H^+]}+1} - \frac{k_b\frac{K_A[P_pH]}{[H^+]}}{\frac{K_A}{[H^+]}+1} =$$

$$= \frac{k_fP_h^0}{\frac{K_A}{[H^+]}+1} - (\frac{k_f\left(\frac{K_A}{[H^+]}+1\right)}{\frac{K_A}{[H^+]}+1} + \frac{k_b\frac{K_A}{[H^+]}}{\frac{K_A}{[H^+]}+1})[P_pH] =$$

Finally,

$$\frac{d[P_pH]}{dt} = \underbrace{\frac{k_fP_h^0}{\frac{K_A}{[H^+]}+1}}_{A} - \underbrace{(k_f + \frac{k_b\frac{K_A}{[H^+]}}{\frac{K_A}{[H^+]}+1})}_{k}[P_pH]$$

Similar to Model 1, we have come to the equation of the type

$$\frac{dy}{dt} = A - ky \tag{S16}$$

the same as in equation (6) but the apparent rate constant of the P_pH concentration change will be described now by the expression:

$$k_{apparent} = k_f + \frac{k_b}{\frac{[H^+]}{K_A} + 1} \qquad \text{(S17)}$$

(compare to equation (S9) for Model 1).

Similarly to Model 1, this rate constant has two limit values:

$$\lim_{[H^+]\to 0}\left(k_f + \frac{k_b}{\frac{[H^+]}{K_A} + 1}\right) = k_f + k_b$$

and

$$\lim_{[H^+]\to \infty}\left(k_f + \frac{k_b}{\frac{[H^+]}{K_A} + 1}\right) = k_f$$

As $[H^+]$ increases from 0 to infinity, we should have the following picture, which quite opposite to the dependence we had for Model 1:

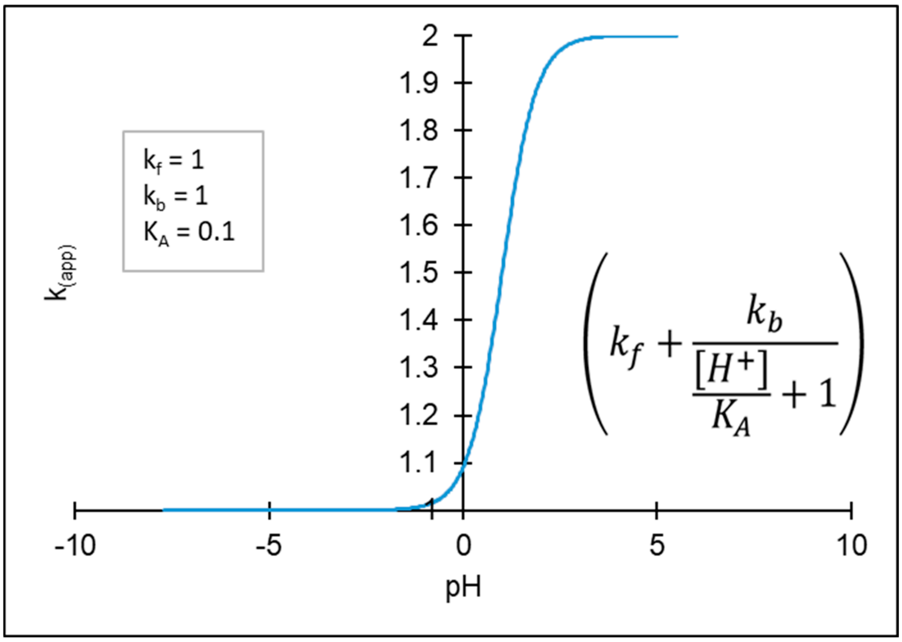

Measurement of the rate of the pentacoordinate form formation under acidic pH jumps allow distinguishing between the two possible models.

Interaction of lipids with the penta-coordinate heme states

(the equilibrium case)

$$P_h \underset{-H^+}{\overset{+H^+}{\rightleftarrows}} P_hH \rightleftarrows P_pH \underset{-L}{\overset{+L}{\rightleftarrows}} LP_pH \qquad \text{(S18)}$$

The starting form of the protein (P) is hexacoordinate (subscript h). It is in equilibrium with its protonated form P_hH which can spontaneously, and reversibly, undertake a conformational change into a pentacoordinate form P_pH. Lipids (L) can bind only that state and reversibly form LP_pH – lipid bound protonated pentacoordinate protein.

In the equilibrium, the three reversible reactions are characterized by the equilibrium constants K_A (protonation/deprotonation), K_C (conformational hexa/penta change) and K_L (lipid binding/unbinding):

$$P_h \underset{K_A}{\overset{+H^+}{\underset{-H^+}{\rightleftarrows}}} P_hH \underset{K_C}{\rightleftarrows} P_pH \underset{K_L}{\overset{+L}{\underset{-L}{\rightleftarrows}}} LP_pH$$

These equilibrium constants can be formulated as follows via the equilibrium concentrations of the four protein forms:

$$K_A = \frac{[P_h][H^+]}{[P_hH]} \tag{S19}$$

$$K_C = \frac{[P_pH]}{[P_hH]} \tag{S20}$$

$$K_L = \frac{[P_pH][L]}{[LP_pH]} \tag{S21}$$

Note that all concentrations presented in square brackets in (S19)-(S21) are *equilibrium* concentrations. We now define the fraction of the protein in the pentacoordinate state as Y. For the equilibrium state,

$$Y = \frac{[P_pH] + [LP_pH]}{[P_pH] + \left[LP_pH\right] + [P_h] + [P_hH]} \tag{S22}$$

The four states in S22 can be substituted using relationships (19)-(21) so that Y will be expressed as a function of the proton concentration $[H^+]$ and lipid concentration $[L]$, with the equilibrium constants K_A, K_C and K_L as parameters:

$$Y = \frac{\left(1 + \frac{[L]}{K_L}\right)[H^+]}{\frac{K_A}{K_C} + \left(1 + \frac{1}{K_C} + \frac{[L]}{K_L}\right)[H^+]} \tag{S23}$$

This can be transformed to an equation useful in experimental monitoring of Y as function of the proton concentration, for a given lipid concentration $[L]$:

$$Y = \frac{A[H^+]}{B + [H^+]} \tag{S24}$$

where

$$A = \frac{1}{1 + \frac{K_L}{K_C(K_L + [L])}} = \frac{K_C(K_L + [L])}{K_C(K_L + [L]) + K_L} = \frac{K_C(1 + \frac{[L]}{K_L})}{1 + K_C(1 + \frac{[L]}{K_L})} \tag{S25}$$

and

$$B = \frac{K_A K_L}{K_C(K_L + [L]) + K_L} = \frac{K_A}{1 + K_C\left(1 + \frac{[L]}{K_L}\right)} \tag{S26}$$

When $[L]$=0, (S25) and (S26) become:

$$A = \frac{K_C}{1 + K_C} \tag{S27}$$

$$B = \frac{K_A}{1 + K_C} \tag{S28}$$

It follows from (S24) that $Y = A$ at infinitely high proton concentration (pH=0) and Y=0 at $[H^+]$=0 ($pH = \infty$). Experimental pH titration of the globin while measuring of the pentacoordinate fraction (optically or by EPR) will give a titration curve with a 50 % transformation (Y=A/2) at $[H^+] = B$. Thus, apparent pK_A of the hexa/penta transformation will be

$$pK_A^{app} = -\log_{10} B \tag{S29}$$

In a similar way, we can transform (S23) for convenient following Y in a titration with lipids experiment (at a given pH)

$$Y = \frac{C + [L]}{D + [L]} \tag{S30}$$

where

$$C = K_L \tag{S31}$$

and

$$D = K_L + \frac{K_L}{K_C}\left(1 + \frac{K_A}{[H^+]}\right) = K_L(1 + \frac{1}{K_C} + \frac{K_A}{K_C}\frac{1}{[H^+]}) \tag{S32}$$

In an experiment when the globin is titrated with lipids, at the starting point (when $[L]$=0), Y is not zero but a simple function of the proton concentration:

$$Y = \frac{C}{D} = \frac{1}{1 + \frac{1}{K_C} + \frac{K_A}{K_C}\frac{1}{[H^+]}} \qquad \text{(S33)}$$

The two limit cases of (S33), i.e. at zero and infinite proton concentrations, before any lipid is added, give the following ((S34) and (S35)):

$$Y_{pH=\infty} = \lim_{[H^+]\to 0} \frac{1}{1 + \frac{1}{K_C} + \frac{K_A}{K_C}\frac{1}{[H^+]}} = 0 \qquad \text{(S34)}$$

…basically meaning that when there is no lipids and no protons, the globin is 100 % hexacoordinate (Y=0).

$$Y_{pH=0} = \lim_{[H^+]\to \infty} \frac{1}{1 + \frac{1}{K_C} + \frac{K_A}{K_C}\frac{1}{[H^+]}} = \frac{1}{1 + \frac{1}{K_C}} = \frac{K_C}{1 + K_C} \qquad \text{(S35)}$$

…which is exactly the *A* value in the absence of lipids (see S27), the maximal possible pentacoordinate fraction, as follows from the equilibrium constant of internal conformations change K_C, when all of the globing is protonated.
